# Prophylaxis by doravirine-lamivudine-tenofovir disoproxil fumarate or elvitegravir-cobicistat-emtricitabine-tenofovir alafenamide after sexual exposure to HIV

**DOI:** 10.1186/s12879-023-08544-x

**Published:** 2023-09-04

**Authors:** Inès Devred, Kick Kayembe, Nadia Valin, Hayette Rougier, Bruce Wuembulua Shinga, Sidonie Lambert-Niclot, Thibault Chiarabini, Marie-Caroline Meyohas, Karine Lacombe

**Affiliations:** 1 Hôpital Saint-Antoine, Assistance Publique Des Hôpitaux de Paris, Service Des Maladies Infectieuses Et Tropicales, 184 Rue du Faubourg Saint-Antoine, F75571, Cedex 12 Paris, France; 2 Institut de Médecine et d’Epidémiologie Appliquée, Paris, 75018 France; 3 Sorbonne Université, 91-105 Boulevard de L’Hôpital, 75013 Paris, France; 4 Hôpital Saint-Antoine, Assistance Publique Des Hôpitaux de Paris, Service de Virologie, 184 Rue du Faubourg Saint-Antoine, F75571, Cedex 12 Paris, France; 5 Institut Pierre Louis de Santé Publique, Inserm UMR-S1136, F75571, Cedex 12 Paris, France

**Keywords:** Post-exposure prophylaxis, Doravirine, HIV, Adherence, Completion rate

## Abstract

HIV post- exposure prophylaxis (PEP) is a prevention tool for individuals with a recent potential exposure to HIV. Doravirine has been available since 2019 in combination with tenofovir disoproxil fumarate and lamivudine and has not been evaluated as a PEP. DOR/3TC/TDF is our department’s most commonly prescribed PEP treatment since 2021. This study evaluates the completion rate of the DOR/3TC/TDF as compared to EVG/c/FTC/TAF for PEP, which was the regimen prescribed until 2020 in our hospital.

This retrospective observational study was conducted between January 2020 and September 2021. The subjects included consecutively were adults who consulted for an HIV sexual exposure accident and for whom DOR/3TC/TDF in 2021 or EVG/c/FTC/TAF in 2020 was prescribed. The outcomes were the completion rate to the end of treatment (28 days), the seroconversion rate, and the description of side effects.

During the study period, 311 people were included: 140 treated with DOR/3TC/TDF and 171 treated with EVGc/FTC/TAF. Considering subjects with a follow-up visit, the completion rate was 96.8% (90/93) in the DOR/3TC/TDF group, and 94.6% (123/130) in the EVG/c/FTC/TAF group (*p*-value: 0.53). The number of people lost to follow-up was nearly equivalent in both groups: 27.1% (38/140) in the DOR/3TC/TDF group and 23.4% (40/171) in the EVG/c/FTC/TAF group (*p*-value: 0.45). A side effect was described for 38% (36/94) in the DOR/3TC/TDF group, and 29.7% (38/128) in the EVG/c/FTC/TAF group. No cases of seroconversion were observed.

DOR/3TC/TDF appears to have a similar safety profile to EVG/c/FTC/TAF. Due to its lower cost, it seems to be a treatment option for consideration in the context of HIV-exposure accidents.

## Background

HIV post- exposure prophylaxis (PEP) is a prevention tool for individuals with a recent (< 48h according French recommendation [1]) potential exposure to HIV. PEP regimens comprise three antiretroviral drugs, and the preferred regimen primarily emphasizes safety and tolerability that should improve treatment acceptability and adherence through the 28-day period. Currently, PEP guidelines in the United States and Europe recommend tenofovir disoproxil fumarate (TDF) and emtricitabine (FTC) as the preferred backbone drugs with a protease inhibitor (PI) or an integrase inhibitor (II), as the third drug [1–3].

French guidelines (2017) recommend PEP in case of vaginal/anal insertive/receptive intercourse or in case of receptive oral sex with ejaculation, with a partner whose viral load is > 50 copies/ml or a partner whose seropositive status is not known, but who belongs to a high seroprevalence group. The regimen of choice according to French guidelines since 2017 is TDF/FTC with rilpivirine [1], but these guidelines have not been updated, and we note that most French hospitals use a combination with an anti-integrase.

The combination of tenofovir alafenamide-emtricitabine with elvitegravir-cobicistat was mainly prescribed in our department until 2020. This treatment, available as one pill once daily, has good tolerability and a high antiretroviral potency but is expensive and contains a booster (cobicistat) that can cause multiple interactions [4]. The approval of new antiretroviral drugs such as doravirine led us to revise local recommendations.

Doravirine (DOR) is a recent antiretroviral drug belonging to the non-nucleoside reverse transcriptase inhibitor (NNRTI) class, available since 2019 in fixed drug combination with tenofovir disoproxil fumarate and lamivudine (DOR/3TC/TDF, Delstrigo®) [5], and has been our department’s most commonly prescribed PEP treatment since early 2021. Doravirine exhibits a reassuring profile of resistance and tolerability [6], close to what has been reported with rilpivirine/3TC/TDF, recommended in the French HIV Post-Exposure Guidelines [1]. It can be taken with or without food and contains no booster. Finally, a doravirine-based PEP has the most reasonable price compared with others (442.76 euros for Delstrigo® compared with 882.16 euros for Genvoya® in France). Currently, the doravirine-lamivudine-tenofovir (DOR/3TC/TDF) combination has not been evaluated as a PEP treatment. The study presented herein was designed to evaluate the acceptability and tolerability of the DOR/3TC/TDF single-tablet regimen as compared to EVG/c/FTC/TAF for HIV PEP.

## Methods

### Participants, setting, data collection, and outcome

This retrospective observational study was conducted in the infectious diseases department of the Saint-Antoine Sorbonne University Teaching Hospital between January 1, 2020 and September 30, 2021. The subjects were included consecutively; they were HIV-negative men and women over 18 years of age who were seen following accidental sexual exposure to HIV and were prescribed a treatment with EVG-150 mg/COBI-150 mg/FTC-200 mg/TAF-10 mg (Genvoya®) or 3TC-300 mg/TDF-245 mg/DOR-100 mg (Delstrigo®). Subjects who were receiving PEP other than EVG/c/FTC/TAF in 2020 or DOR/3TC/TDF in 2021 were not included.

After a potential sexual risk exposure, individuals usually visit the Teaching Hospital emergency department where PEP is systematically prescribed for 5 days. Indications are then re-assessed during a visit at the infectious diseases department within those 5 days. Finally, at a third follow-up visit 6–8 weeks later, the practitioner assesses compliance and tolerability of PEP, collects follow-up serology, and discusses further management. Individuals received a reminder call in case of a missed week-8 appointment. Blood testing was prescribed on day 0 to assess HIV serologies, AST/ALT, creatinemia, and β-HCG (for women); on day 15 to assess biologic tolerance for individuals with comorbidity or in the case of fear of an iatrogenic effect (AST/ALT, creatinemia); and at week 8 to assess HIV serologies, as recommended [1].

Between January 1, 2020 and December 31, 2020, practitioners mainly prescribed EVG/c/FTC/TAF, and from January 1, 2021 onwards, practitioners changed their treatment habits and prescribed DOR/3TC/TDF, according to local recommendations. The following data were extracted from the Diamm/G 8.7 consultation software: age, previous PEP, partner’s HIV status, time of PEP initiation, type of sex, condom use (initial visit); treatment completed through day 28, regularly taking or forgetting tablet (every day / almost every day / occasionally / rarely), side effects, type of side effects, impact on quality of life (no / moderate / significant / major discomfort), and serologies (follow-up visit). The PEP adherence through the 28-day period, which is our primary outcome, was assessed through self-reporting with the question “Did you complete the treatment until the end of the 28 days?”.

Subjects were individually informed that their medical data could be used for research purposes and signed an information note in accordance with the General Data Protection Regulation. The study protocol was approved by the institutional review boards of Saint-Antoine Hospital and Sorbonne University (registration number with the Data Protection Committee: 20,211,228,115,250).

### Data analysis

The main outcomes were PEP adherence through the 28-day period, and occurrence of side effects reported by the subjects. We also reported any HIV seroconversion, and the subjects also reported the impact of PEP on quality of life due to side effects. Per-protocol analysis was performed, including subjects with a follow-up visit or telephone contact. For statistical analysis, the completion and side effect rates were expressed as a percentage, with 95% confidence intervals. A chi-square test was used to identify significant differences between the two treatment groups. The alternative Fisher’s exact t-test was preferred if the conditions of validity were not met for a chi-square test. *P* < 0.05 was considered statistically significant.

## Results

### Baseline characteristics and follow-up of subjects

During the study period, 311 people were included overall: 140 treated with DOR/3TC/TDF and 171 treated with EVG/c/FTC/TAF (Table 1). The majority of subjects were male (85.2%). The median (range) age was 30 years (18–75 years). Most of the male subjects were men who have sex with men (77.3%). No subject was excluded because of their HIV-positive status.

**Table 1:** Characteristics of exposed individuals at the initial visit

	EVG/c/FTC/TAF	DOR/3TC/TDF	*p*-value
	*n* = 171	*n* = 140	
Age, median (min–max)	30 (18–75)	29 (18–70)	0.80
Male	150 (87.7%)	115 (82.1%)	0.17
Previous HIV non-occupational exposure	64 (37.4%)	31 (22.1%)	0.03
HIV-infected source subject	8 (4.7%)	6 (4.3%)	1
Previous sexually transmitted diseases	27 (15.8%)	29 (20.7%)	0.26
Condom:			
No condom use	85 (50.3%)	77 (56.6%)	
Slippage of the condom	14 (8.3%)	4 (2.9%)	
Condom breakage	70 (41.4%)	53 (39.0%)	
Use of condom	0	2 (1.5%)	
Type of exposure: MSM (men who have sex with men)	110 (74.3%)	91 (81.3%)	0.23
Use of chemsex	18 (10.5%)	14 (10.0%)	0.87
Type of sex:			
Insertive anal	32 (18.9%)	43 (31.6%)	
Receptive anal	70 (41.4%)	47 (34.6%)	
Insertive oral sex	3 (1.8%)	0	
Receptive oral sex	7 (4.1%)	6 (4.3%)	
Receptive or insertive vaginal	53 (31.4%)	39 (28.7%)	
Others	4 (2.4%)	1 (0.7%)	

**Fig. 1:**
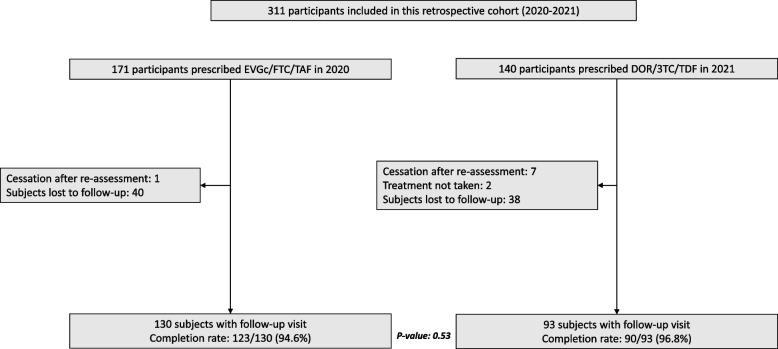
Flow chart of participants who consulted after sexual exposure to HIV and were prescribed EVG/c/FTC/TAF in 2020 or DOR/3TC/TDF in 2021

Among the 311 subjects, a medical reevaluation led to an early discontinuation of treatment for 8 subjects (7 in the DOR/3TC/TDF group and 1 in the EVGc/TAF/FTC group) because we learned after the infectious disease consultation that the source subject was finally not infected with HIV or that his viral load was undetectable, 2 subjects in the DOR/3TC/TDF group never started PEP and therefore were not considered for the completion rate, and 78 individuals were considered lost to follow-up because they did not attend the follow-up consultation and could not be reached by telephone. The per-protocol analysis included therefore 216 subjects: 93 in DOR/3TC/TDF group and 123 in EVGc/TAF/FTC group (Fig. 1).

### Completeness and seroconversion rate

In the DOR/3TC/TDF group, the completeness rate was 96.8% (90/93, 95% CI [93.2%–100%]) in per-protocol analysis. Among subjects who did not complete their treatment, 2 interrupted PEP due to misunderstanding and one due to a poor tolerability (ocular reaction after 15 days). In the EVGc/FTC/TAF group, the completion rate was 94.6% (123/130, 95% CI [92.7–98.4%]) in per-protocol analysis. Among the 7 subjects who did not complete their treatment, 5 had poor treatment tolerability (diarrhea, rash with increased level of AST/ALT, and not specified), 2 subjects expressed they misunderstood the reason for PEP. The two treatment groups were comparable in terms of completion rate (*p*-value: 0.53 using the Fisher’s exact test) (Fig. 1). Regarding adherence, among the subjects who completed DOR/3TC/TDF, 89.8% (79/88) answered that they took the treatment every day, and 10.2% (9/88) answered that they took the treatment almost every day. Among the subjects who completed the EVG/c/FTC/TAF treatment, 93.4% (114/122) answered that they took the treatment every day, and 6.6% (8/122) answered that they took the treatment almost every day.

No cases of seroconversion were observed in the two treatment groups among the 191 subjects (61.4%) who reported having a follow-up serological assessment.

### Occurrence of side effects

The cumulative incidence of side effects over the treatment period was estimated at 33.3% (77/222) of the subjects and was not significantly different between the two groups (*p*-value = 0.11): 38.3% (36/94) of subjects receiving DOR/3TC/TDF and 29.7% (38/128) of subjects receiving EVGc/TAF/FTC (Table 2). Most of the side effects were digestive (nausea, vomiting, and diarrhea) or fatigue. Other rarer effects have been reported with DOR/3TC/TDF: rash (1), mucosal damage (1), headache (1), myalgia (1), sleep disorders (1), dysgueusia (1), and hypersalivation (1). Three subjects who received EVGc/TAF/FTC showed minor liver toxicity. The impact of side effects on daily life was significant for 3.4% of subjects receiving DOR/3TC/TDF and 4% for EVGc/TAF/FTC.

**Table 2:** Characteristics of side effects

	EVG/c/FTC/TAF	DOR/3TC/TDF	*p*-value
	*n* = 128	*n* = 94	
Occurrence of at least one side effect, n (%)	38 (29.7%)	36 (38%)	0.11
Discontinuation of treatment due to poor tolerance	5 (3.9%)	1 (1.1%)	0.40
Clinical side effects			
Digestive side effect: nausea or vomiting n (%)	14 (10.1%)	13 (13.8%)	
Digestive side effect: diarrhea, n (%)	8 (6.2%)	8 (8.5%)	
Rash	2 (1.6%)	1 (1.1%)	
Mucous membrane lesions	0	1 (1.1%)	
Headache	2 (1.6%)	1 (1.1%)	
Fatigue, discomfort	2 (1.6%)	5 (5.3%)	
Myalgia	0	1 (1.1%)	
Sleep disorders, anxiety	0	1 (1.1%)	
Dysgueusia	0	1 (1.1%)	
Hypersalivation	0	1 (1.1%)	
Increase in AST and ALT	3 (2.3%)	0	
Not specified	6 (4.7%)	9 (9.6%)	
**Impact on daily life, n**	***n* = 125**	***n* = 89**	
No discomfort, n (%)	103 (82.4%)	67 (75.3%)	
Moderate discomfort, n (%)	16 (12.8%)	19 (21.3%)	
Significant discomfort, n (%)	5 (4%)	3 (3.4%)	
Major discomfort, n (%)	1 (0.8%)	0	

### Individuals lost to follow-up

The rate of lost to follow-up was 25.1% (78/311) and was similar between the two groups: 27.1% (38/140) in DOR/3TC/TDF group versus 23.4% in EVGc/TAF/FTC group (*p*-value: 0.45). Baseline characteristics (age, sex, previous PEP, history of sexually transmitted infection, type of sex, regular partner, usual condom use) did not significantly differ between lost to follow-up individuals and follow-up individuals.

## Discussion

Completion of 28 days of HIV post-exposure treatment is a major challenge to its effectiveness. This study, evaluating the acceptability and safety of doravirine-based PEP (TDF/3TC/DOR) as one pill, reported a 96.8% completion rate compared to 94.6% for EVG/c/FTC/ TAF previously used in our department (*p*-value: 0.53), considering only subjects followed to term (233/311 subjects). These rates are similar to the rates currently found in the literature with single-tablet regimens based on integrase inhibitor or rilpivirine. Foster et al. investigated a single-tablet PEP regimen based on rilpivirine and estimated a completion rate of 92% [7]. Valin et al. estimated a completeness rate > 95% with elvitegravir, cobicistat, FTC, and TDF among subjects with follow-up [8]. Gantner et al. evaluated prospectively EVG/c/FTC/TAF and found an 82% completion rate, including those lost to follow-up [9]. Raltegravir (RAL/FTC/TDF) and dolutegravir (DTG/TDF/FTC) also showed completion rates above 90% [10, 11]. A network analysis seems to show a higher completion rate of integrase inhibitor combinations than previous strategies with protease inhibitors [12]. Recently, co-formulated BIC/FTC/TAF was found to be safe and well tolerated, with a 90.4% completion rate [13], and could be a future PEP option, particularly in people with renal failure.

Side effects were reported slightly more frequently in the DOR/3TC/TDF group (38% of subjects) than in the EVG/c/FTC/TAF group (29.7%), but this difference was not significant (*p*-value: 0.11). As expected, digestive side effects, namely nausea and diarrhea, predominated. The prevalence of side effects was lower than in other studies with elvitegravir. Gantner et al. (evaluating EVGc/FTC/TAF) found side effects in 68% and 59% of subjects at the day-14 and day -8 visits, respectively [9]. In our study, only 29.7% of subjects receiving EVGc/TAF/FTC had side effects. This prevalence may have been underestimated due to the retrospective nature of our study and because the follow-up visit took place after the end of treatment. The absence of a systematic lab test at day 15 may also have led to an underestimation of cases of increased liver enzymes or renal failure.

The financial cost of exposure accidents is high and should be considered in the choice of treatment, if the molecules seem comparable in terms of adherence. The public price in France of a 30-tablet box of DOR/3TC/TDF (Delstrigo®) currently costs 442.76 euros, whereas the combination EVG/c/FTC/TAF (Genvoya®) costs 882.16 euros. Other rilpivirine-based combinations cost 492.24 euros. Another point to be discussed is PEP’s appropriateness in the context of sexual risk behaviors: Is PEP prescribed appropriately [14] or is it over-prescribed? Given the multiplication of risky sexual behaviors and the rise of chemsex practices in France and Europe [15], HIV prevention tools are needed, and the number of PEP prescriptions could increase in the coming years.

Doravirine is not only the most reasonably priced PEP, its characteristics such as a satisfying genetic barrier and low risk of drug interaction make it one of the preferred treatments for HIV post-exposure. Doravirine-based PEP could also be a good alternative particularly because of its good oral bioavailability, the absence of any food constraint, and its penetration into the deep compartments (24% free fraction available). In France, the majority of PEP prescribed for HIV is the combination of emtricitabine-rilpivirine-tenofovir disoproxil fumarate (Eviplera®) [16] or single-tablet regimens based on integrase inhibitor. Rilpivirine is sensitive to intestinal pH fluctuations with antacids, whereas doravirine is not. Moreover, the prevalence of transmitted resistance in antiretroviral treatment-naive subjects is significantly higher for rilpivirine (8.1%) than for doravirine (1.4%) [17–19] notably because of the polymorphic mutation E138K, which impacted RPV susceptibility. Thus, the prescription of a doravirine-based PEP regimen seems adapted to the French epidemiological situation. In any case, local recommendations should take into account the prevalence of transmitted resistance in antiretroviral treatment-naive patients. Doravirine-based PEP may not be appropriate in geographical areas with a high degree of resistance mutations compared to PI-based or second-generation integrase II-based regimens.

In this study and in our practices, we decided to change to TDF use (DOR/3TC/TDF), instead of TAF used before 2021 (EVG/c/FTC/TAF). Although there are higher risks of bone and renal adverse events with TDF compared with TAF [20], it does not appear that this toxicity can develop so rapidly during PEP treatment [21]. However, a treatment option containing TAF rather than TDF is preferable in subjects with renal failure.

In this study, no seroconversion was observed. However, it seems difficult to assess the effectiveness of post-exposure treatment because we are often unable to determine the HIV-positive status and viral load of the initial source subject, and particularly in this retrospective study because of the high rate of subjects lost to follow-up.

This study’s main limitation is the number of individuals lost to follow-up and information bias (underestimating side effects) due to the retrospective nature of data collection. The number of lost to follow-up, including individuals who missed their follow-up appointment was really high and appears to be higher than in others studies [8, 9]. However, these data reflect real life and seem to correspond to a high rate of absenteeism in the follow-up of PEP treatment, even outside the context of COVID. Secondly, adherence is self-reported (no pill count, no use of a smart pillbox as a medication event monitoring system, no drug plasma level, etc.). This may overestimate the completion rate, as subjects may tend to hide a lack of compliance. Third, although we did not report any cases of seroconversion, the study was not designed to assess the effectiveness of PEP. As with this one, the majority of studies on PEP investigate the completion rate since it is very rare to observe seroconversion after PEP treatment. Furthermore, the comparison between the two treatment groups was reconstructed a posteriori; since prescribing habits have changed, it is obviously a non-randomized comparison. The lack of a systematic lab test is indeed a deficiency of the study. A prospective study tracking side effects in a more systematic way should supplement this study to ensure that this treatment is well tolerated, and a larger study including occupational exposure would also be interesting since subjects may differ when comparing sexual or occupational exposure to HIV.

## Conclusion

This retrospective study is the first, to our knowledge, to evaluate DOR/3TC/TDF as a post-exposure treatment. DOR/3TC/TDF treatment appears to have a good completion rate and a similar safety profile to EVG/c/FTC/TAF. Due to its low cost, good tolerance without food intake constraints, and low potential for cross resistance to didanosine, it seems to be a treatment option for consideration in the context of HIV-exposure accidents.

## Data Availability

The datasets analyzed in this study are available from the corresponding author upon reasonable request.
